# High Dietary Supplementation of Procyanidin-Rich Grape Seed Powders Enhances the Growth Performance and Muscle Crispness of Crisped Grass Carp

**DOI:** 10.3390/ani16020251

**Published:** 2026-01-14

**Authors:** Ziqiu Peng, Qiuwen Tang, Haojun Liang, Xiaoyi Zhang, Xiaoye Wang, You Li, Ping Ding, Yongzhan Mai, Xuesong Wang

**Affiliations:** 1 Key Laboratory of Chinese Medicinal Resource from Lingnan, Ministry of Education, Institute of Medical Plant Physiology and Ecology, School of Pharmaceutical Sciences, Guangzhou University of Chinese Medicine, Guangzhou 510006, China; 2 Guangdong Provincial Key Laboratory of Chemical Measurement and Emergency Test Technology, Guangdong Provincial Engineering Research Center for Ambient Mass Spectrometry, Institute of Analysis, Guangdong Academy of Sciences (China National Analytical Center, Guangzhou), 100 Xianlie Middle Road, Guangzhou 510070, China; 3 School of Chemistry, Chemical Engineering and Materials Science, Shandong Normal University, Jinan 250014, China; 4 Scientific Observing and Experimental Station of Fishery Resources and Environment in the Middle and Lower Reaches of Pearl River, Key Laboratory of Prevention and Control for Aquatic Invasive Alien Species, Fishery Ecological Environment Monitoring Center of Pearl River Basin, Ministry of Agriculture and Rural Affairs, Guangdong Provincial Key Laboratory of Aquatic Animal Immunology and Sustainable Aquaculture, Pearl River Fisheries Research Institute, Chinese Academy of Fishery Sciences, Guangzhou 510380, China

**Keywords:** crisped grass carp, grape seed powder, growth performance, muscle crispness, intestine microbiota

## Abstract

A comprehensive analysis was conducted to explore whether feeding inclusion procyanidin-rich grape seed powders (GSPs) affected the faba bean-induced muscle crispness in the aquaculture of crisped grass carp. The procyanidin content in the prepared GSP was 10.40 g/100 g. Additionally, one thousand 1-year-old grass carp with an initial weight of 27 g and an initial length of 12 cm were divided into five groups, including the blank control (basal diet); the positive control (faba bean diet); and the low (faba bean diet supplemented 100 mg/kg GSP), middle (faba bean diet supplemented 500 mg/kg GSP), and high (faba bean diet supplemented 1000 mg/kg GSP) GSP-supplemented groups. After feeding for 60 days, the weight gain rate, specific growth rate, and condition factor were elevated in the high-GSP-supplemented group in comparison with the blank control (*p* < 0.05), accompanied by a significant decrease in the feeding coefficient (*p* < 0.05). Meanwhile, a significant increase in muscle ROS content, shear force, gumminess, and chewiness was determined in the high-GSP-supplemented group when compared with the positive group, suggesting that a relatively high daily supplement of GSP facilitated muscle crispness. Moreover, the composition of intestine microbiota was significantly varied between groups with the daily addition of GSP (*p* < 0.05). Among them, *Lactococcus chungangensis* was identified as the key biomarker of the high-GSP-supplemented group, which was closely related to the increased muscle ROS content, the modifications in muscle nutritional metabolites (Met, C20:2n6, C20:3n6, C20:4n6, and C22:4n6), and the alterations in muscle texture (gumminess, chewiness, shear force, hardness, and adhesiveness). Based on these results, we believe that a relatively high daily supplement of GSP (1000 mg/kg) facilitated muscle crispness in the aquaculture of crisped grass carp.

## Simple Summary

The muscle crispness of crisped grass carp is triggered by the faba bean-induced ROS reaction. Grape seed powders, which potentially serve as an alternative to antibiotics in the healthful aquaculture of crisped grass carp, are limited to being used as free radical scavengers. In this study, a relatively high daily supplement of procyanidin-rich grape seed powders (GSPs) enhanced the growth performance of crisped grass carp and facilitated muscle crispness, which proved that procyanidin-rich GSPs could replace antibiotics in the aquaculture of crisped grass carp.

## 1. Introduction

Grass carp (*Ctenopharyngodon idella*) holds the leading position in aquaculture due to its worldwide production that surpasses six million tons [1]. Meanwhile, grass carp is the principal commercial fish of China [2], and the total quantity of grass carp products in China has reached 5.57 × 10^6^ tons, which is approximated to be 80% of the global production [3]. Remarkably, the crisped grass carp is particularly famous in South China due to its unique crunchy texture, and the annual output value of crisped grass carp has approached CNY 1.5 × 10^8^ [4]. In the comparative analysis of the muscle between the crisped grass carp and the conventional variety, apparent differences in the muscle texture are revealed, exhibiting as the increased shear force, hardness, gumminess, chewiness, and springiness in crisped grass carp [5]. In the aquaculture of crisped grass carp, faba bean (*Vicia faba*) is absolutely needed, and the daily supplementation of faba bean for 90–120 days contributes to muscle crispness [6]. A well-recognized view is that the dietary addition of faba bean facilitates reactive oxygen species (ROS) elevation and subsequently produces in vivo oxidative stress, which disrupts Ca^2+^ binding with actin, as well as actin’s interaction with myosin [1]. Therefore, muscle crispness is well associated with the faba bean-induced ROS reaction.

To meet the economic and dietary demands, the healthful aquaculture of crisped grass carp is necessary. The literature reports that the growth performance of crisped grass carp is severely affected by typical diseases, such as ichthyophthiriasis and reovirus [7,8]. Prior to 1 July 2020, antibiotics were commonly employed to maintain the massive production of aquaculture [9]; however, since then, the Chinese government has prohibited the application of antibiotics due to their detrimental effects on humans via the consumption of these aquaculture products, such as genetic, cell, and reproduction toxicities [10,11]. Consequently, new alternatives to antibiotics are urgently needed, and Chinese medical plants are widely utilized in aquaculture based on their merits of being green, safe, and efficient [12,13]. For example, feeding inclusion *Eucommia ulmoides* extract [4] promotes growth in the aquaculture of grass carp. Considering these considerations, the incorporation of plant-based ingredients into diets was potentially conducive to the healthful aquaculture of crisped grass carp.

The grape *Vitis vinifera* L., possessing both edible and medicinal characteristics (e.g., pulp and seed), has been utilized as a traditional medicinal material for thousands of years in China [14], and it exhibits numerous pharmacological and clinical effects [15,16]. Presently, grape seed has received considerable attention owing to the identification of its various plant-based bioactive constituents, such as polyphenols, flavonoids, and procyanidins [17]. Notably, the procyanidin content in Kyoho grape seed was the highest among forty-four evaluated varieties [18]. As a rich source of natural bioactive ingredients, grape seed reveals multiple health benefits, including antiviral, anti-inflammatory, antioxidant, hepatoprotective, cardioprotective, and neuroprotective effects [19,20,21]. For instance, the literature has demonstrated that grape seed possesses ROS-scavenging capacities through the regulation of enzymatic polymerization (such as superoxide dismutase and catalase) and the direct neutralization of free radicals [22]. Furthermore, evidence has shown that the use of grape seed is growth-promoting and disease-preventing in the poultry and livestock industries [23], suggesting its potential for serving as a feed additive in animal farming. However, the application of GSP in aquaculture remains limited. Therefore, whether dietary supplementation with grape seed enhances growth performance and mitigates muscle crispness in the aquaculture of crisped grass carp has attracted our attention, especially the daily supplementation of procyanidin-rich grape seed.

In this study, the effects of dietary supplementation with procyanidin-rich grape seed powders (GSPs) on the growth performance and muscle crispness of crisped grass carp were investigated for the first time. The basal composition of the prepared GSP was analyzed prior to feeding. Subsequently, the growth parameters, physiological/biochemical indexes, muscle amino acids, muscle fatty acids, muscle texture, tissues histology inspection, and intestine microbiome were analyzed at the same time. We hypothesized that feeding inclusion procyanidin-rich GSP was growth-promoting without affecting muscle crispness in the aquaculture of crisped grass carp. Our study could provide new insights for the application of procyanidin-rich GSP in aquaculture.

## 2. Materials and Methods

### 2.1. Preparation of GSP

The Kyoho grape seeds were supplied by South China Agricultural University (Guangzhou, China). After being washed three times with deionized water, the grape seeds were dried in an oven at 40 °C for 1 w. Subsequently, the dried grape seeds were ground using a cyclone mill and sieved with a 90-mesh sieve. The prepared GSP then underwent compositional analysis. The content of procyanidin was quantified following GB/T 22244-2008 [24]. Additionally, the levels of dichlorodiphenyl trichloroethane (DDT), hexachlorocyclohexane (HCH), and quintozene (PCNB) were determined in accordance with GB/T 5009.19-2003 [25] and GB/T 5009.136-2003 [26], respectively. Moreover, the contents of lead (Pb), cadmium (Cd), mercury (Hg), and arsenic (As) were assessed following the standard of “Determination of multielement in food”, National Health Commission (NHC) [27]. The microbiological analyses, including Salmonella, *Staphylococcus aureus*, aerobic bacterial counts, molds and yeasts, and coliforms, were conducted according to the standards of “Determination of *Salmonella* spp.”, “Determination of *Staphylococcus aureus*”, “Determination of total bacterial count”, “Determinaton of mould and yeast count”, and “Determination of coliform count”, respectively [28,29,30,31,32].

### 2.2. Animal Trials

One-year-old grass carp (average body weight of 27 g and average body length of 12 cm) were selected for animal trials, which were purchased from Guangzhou Fulong Aquaculture Company, Guangzhou, China. One week of acclimation was performed for all fish in 50 L tanks (48.7 cm × 34.3 cm × 25.8 cm) without feeding. After acclimation, a total of 1000 fish were randomly divided into five groups: a basal diet (blank control, group CK), a faba bean diet (positive control, group VN), a faba bean diet supplemented with 100 mg/kg GSP (low-GSP-supplemented group, group VL), a faba bean diet supplemented with 500 mg/kg GSP (middle-GSP-supplemented group, group VM), and a faba bean diet supplemented with 1000 mg/kg GSP (high-GSP-supplemented group, group VH). In this study, two rules were adopted to set the dose for incorporation: (I) the content of feed additive in the final feed pellets should be lower than 1000 mg/kg, and (II) a tenfold difference (at least) was necessary between the low dose and the high dose. Meanwhile, to have a better comparison, a middle dose was set at the same time. Of these groups, groups VL, VM, and VH were collectively referred to the GSP-supplemented groups. Each group comprised 200 fish, with 50 fish housed in one tank. The basal diet was supplied by Tongwei Group (Chengdu, Sichuan Province, China) (Table S1), while the faba bean diet was supplied by the professional farmers (Qujing, Yunnan, China) (Table S2). For compound feed pellets preparation, the faba bean diet and GSP were sieved through a 200-mesh stainless steel screen and homogenized completely. Subsequently, sterile water was added in a ratio of 4:1 (weight to volume), and the obtained mixture was re-pelletized using a stainless steel feed pellet machine. The pellets were then dried at 56 °C for 1 h, so that the moisture content was below 10%. The prepared compound feed pellets were used for animal trials unless otherwise specified. The rearing conditions were maintained consistently during the whole periods of animal trials (Table S3), and the feeding period was 60 days. When animal trials finished, the body weight and body length were recorded individually. Subsequently, all fish were euthanized, and Sigma-Aldrich^®^ (St. Louis, MO, USA) tricaine methanesulfonate was used during euthanasia (MS-222, 5 ppm).

### 2.3. Sample Collection

The gills, intestine, and dorsal muscles were collected from the fish of each group (n = 200 per group). Of these, fifty percent of samples were preserved in 4% paraformaldehyde for histological examination, while the remaining fifty percent were stored at −80 °C for alternative analyses. Additionally, the intestinal contents were harvested (n = 200 per group) and preserved at −80 °C for intestine microbiome sequencing and analysis.

### 2.4. Growth Parameter Calculations

The weight gain rate (WGR, %, Formula (1)), specific growth rate (SGR, %/d, Formula (2)), condition factor (CF, %, Formula (3)), feeding coefficient (FC, %, Formula (4)), hepatosomatic index (HSI, %, Formula (5)), and viscerosomatic index (VSI, %, Formula (6)) were calculated based on a previously published work [12].

WGR = (final body weight − initial body weight)/initial body weight × 100;(1)

SGR = (In (final body weight) − In (initial body weight)) ÷ feeding days × 100;(2)

CF = final body weight/(final body length)^3^ × 100; (3)

FC = feed consumption/(final body weight − initial body weight) × 100;(4)

HIS = final liver weight/final body weight × 100;(5)

VSI = final visceral weight/final body weight × 100;(6)

### 2.5. Histology Inspection

As previously mentioned, paraformaldehyde (4%, *v*/*v*) was used to fix tissue samples, including the gills, intestine, and dorsal muscles (n = 10 per group). Subsequently, the tissues were dehydrated with graded ethanol solutions, followed by clearing with Sigma-Aldrich^®^ xylene and embedding in Sigma-Aldrich^®^ paraffin. Then, the tissue samples were cut using a Leica^®^ microtome (Wetzlar, Germany) so that 5 μm slices were obtained. Additionally, the slices were subjected to solvent deparaffinization in xylene and rehydrated. After being stained with Sigma-Aldrich^®^ hematoxylin and eosin (H&E), the prepared slices were examined and imaged using an ECHO REVOLVE^®^ microscope (San Diego, CA, USA). The length-to-width ratio in the secondary lamellae in gills, the intestine villi length, and the diameters of muscle fibers were calculated using ImageJ software (Version 1.53t, National Institutes of Health, Bethesda, MD, USA).

### 2.6. Physiological and Biochemical Indexes’ Determination

The gills, intestine, and dorsal muscles were subjected to physiological/biochemical analyses (n = 10 per group). These tissues were initially homogenized in ice-cold double-distilled water. The activities of superoxide dismutase (SOD), glutathione S-transferase (GST), catalase (CAT), glutathione peroxidase (GPx), malondialdehyde (MDA), and glutathione (GSH) were determined, and the test kits were provided by Solarbio^®^ colorimetric (Beijing, China). Specifically, the SOD activity was measured based on the inhibition of the reduction of nitroblue tetrazolium by the xanthine/xanthine oxidase system at 550 nm, the CAT activity was determined by monitoring the decomposition of H_2_O_2_ at 240 nm, the GPx activity was assessed via the oxidation of glutathione coupled with NADPH consumption at 340 nm, the GST activity was measured using 1-chloro-2,4-dinitrobenzene (CDNB) as a substrate at 340 nm, the MDA content was quantified by its reaction with thiobarbituric acid (TBA) at 532 nm, and the GSH content was determined using 5,5′-dithiobis-(2-nitrobenzoic acid) (DTNB) as a substrate at 412 nm. The total protein concentration of each tissue homogenate was determined using a BCA protein assay kit (Solarbio, Beijing, China), and all enzyme activities were normalized to the protein content (expressed as U/mg prot or nmol/min/mg prot). Meanwhile, the levels of interleukins (IL-1*β*, IL-2, IL-4, IL-6, and IL-12) and immunoglobulin (IgM) were evaluated using enzyme-linked immunosorbent assay (ELISA) kits (Shanghai Enzyme-linked^®^ colorimetric, Shanghai, China).

### 2.7. Muscle Nutrition Detection

The muscle amino acids and muscle fatty acids were analyzed according to NHC [33] and NHC [34], respectively. For muscle amino acid detection (n = 3 per group, the mixed muscles of 10 individuals), samples from dorsal muscles were subjected to acid hydrolysis using 6 M HCl and then freeze-dried in a vacuum freeze-dryer with flowing nitrogen. After hydrolysis at 110 °C for 22 h, the obtained hydrolysate was dried in vacuum at 40 °C and subsequently mixed with 1 mL sodium citrate buffer at pH 2.2. The mixture was filtered through a 0.22 μm filter, and the supernatant was collected. After being transferred into autosampler vials, instrumental analysis was performed. During analysis, a Thermo Accucore XL C-18 column (2.0 × 100 mm, 4 μm, Waltham, MA, USA) was used for separation. The mobile phase A was methanol, and the total run time was 10 min with a flow rate of 1.2 mL/min. The column temperature was maintained at 30 °C, with an injection volume of 10 μL. A Shimadzu LC-20D HPLC system (Kyoto, Japan) was employed for chromatographic analysis. For muscle fatty acid detection (n = 3 per group, the mixed muscles of 10 individuals), fatty acid methyl esters (FAMEs) were prepared via the transesterification of muscle lipids. Separation and analysis were performed using a Shimadzu GC-2019 gas chromatograph equipped with a flame ionization detector (FID) and an HP-88 capillary column (100 m × 0.25 mm × 0.2 μm film thickness). During the analysis, the oven temperature program was set as follows: initial hold at 140 °C for 5 min, then rapid increase to 240 °C at 4 °C/min, and final hold maintained for 20 min. High-purity helium was used as the carrier gas at a constant flow rate. Fatty acids were identified by comparing retention times with the certified FAME standards.

### 2.8. Muscle Texture Analysis

The muscle texture analysis was conducted using a Universal TA texture analyzer (Tengba Instrument Technology Co., Ltd., Shanghai, China). The shear force was measured using a Warner-Bratzler shear blade (HDP/BSW) under the following conditions: single compression, a pre-test speed of 1 mm/s, a testing speed of 1 mm/s, a post-test speed of 3 mm/s, and a trigger force of 20 gf. Additionally, the texture profile analysis (TPA) was performed using a cylindrical probe (P/36R, diameter 36 mm). The dorsal muscle samples were cut into uniform cubes and equilibrated to room temperature (25 °C) before testing. Subsequently, the TPA detection was conducted under the following conditions: a pre-test speed of 1 mm/s, a testing speed of 1 mm/s, a post-test speed of 1 mm/s, a trigger force of 10 gf, a shape change of 40%, and a time interval of 4 s between the two compressions.

### 2.9. Muscle Safety Evaluation

Considering safety, the essential indexes of muscles were evaluated in accordance with the national food safety standards of China (n = 3 per group, the mixed muscles of 10 individuals). The contents of DDT, HCH, and PCNB were analyzed according to GB/T 5009.19-2003 and GB/T 5009.136-2003, respectively. Moreover, the contents of Pb, Cd, Hg, and As were determined according to NHC (2016) [27], and the presence of foodborne pathogenic microorganisms, including *Salmonella* and *Escherichia coli*, was monitored by adhering to NHC (2016) [28,29,30,31,32].

### 2.10. High-Throughput 16S Ribosomal RNA Gene Sequencing

The DNA was extracted from intestine contents using the TGuide S96 Magnetic Soil/Stool DNA Kit (Tiangen Biotech, Beijing, China). The quality and quantity of the extracted DNA were assessed via agarose gel electrophoresis (AGE). In addition, the DNA concentration and purity were determined on a NanoDrop 2000 UV–Vis spectrophotometer (Thermo Scientific, Wilmington, DE, USA). The full-length 16S rRNA gene was amplified using the paired primers, including the forward primer (27F: AGRGTTTGATYNTGGCTCAG) and the reverse primer (1492R: TASGGHTACCTTGTTASGACTT). The PCR amplification was performed using the KOD One PCR Master Mix (TOYOBO Life Science, Osaka, Japan), starting with an initial denaturation at 95 °C for 2 min, followed by 25 cycles of denaturation at 98 °C for 10 s, annealing at 55 °C for 30 s, extension at 72 °C for 1 min and 30 s, and a final step at 72 °C for 2 min. The obtained PCR amplicons were purified with VAHTSTM DNA Clean Beads (Vazyme, Nanjing, China) and quantified using the Qubit dsDNA HS Assay Kit and Qubit 3.0 Fluorometer (Invitrogen, Thermo Fisher Scientific, Hillsboro, OR, USA). After individual quantification, amplicons were pooled in equimolar amounts. SMRTbell libraries were constructed for the amplified DNA using the SMRTbell Express Template Prep Kit 2.0 (Pacific Biosciences, Menlo Park, CA, USA). The purified SMRTbell libraries, derived from pooled and barcoded samples, were sequenced on a PacBio Sequel II platform (Beijing Biomarker Technologies Co., Ltd., Beijing, China) using the Sequel II binding kit 2.0 (Pacific Biosciences Inc., Menlo Park, CA, USA). The raw data are available with the China National Center for Bioinformation (www.cncb.ac.cn) under the accession number PRJCA045579.

### 2.11. Bioinformatic Analysis

The amplicon sequence variants (ASVs) were generated using DADA2, with counts less than two across all samples being filtered out. The taxonomic annotation of ASVs was performed based on the Naive Bayes classifier within QIIME2, employing the SILVA database (release 138.1) with a confidence threshold of 70%. Alpha diversity (Shannon and Simpson) indexes were calculated to assess the within-group diversity. Beta diversity was evaluated using principal coordinate analysis (PCoA) to determine the degree of similarity between microbial communities from different samples. Additionally, Linear Discriminant Analysis Effect Size (LEfSe) was used to identify significant taxonomic differences between groups, with a logarithmic LDA score threshold of 4.0 for discriminative features. Extreme Gradient Boosting (XGBoost) machine learning was applied to identify potential keystone species and to predict functional profiles; only features with an importance score above 0.1 were visualized. The functional composition of the intestine microbiota was predicted using PICRUSt2. Spearman’s correlation analysis was performed to examine the relationships between the bacterial taxa, the predicted functional profiles, and the muscle enzymatic/nutritional and texture parameters, with significant correlations (Spearman’s *r* > 0.3, *p* < 0.05) illustrated through Sankey plots. The analysis, including alpha diversity, beta diversity, and machine learning, was carried out using the EasyMultiProfiler package within the software RStudio (2023.12.0 + 369).

### 2.12. Statistical Analysis

All data are presented as mean ± standard deviation. Initially, the normality of the data was assessed using the Shapiro–Wilk test. If the data conformed to a normal distribution, a parametric one-way ANOVA combined with Fisher’s Least Significant Difference (LSD) test was employed to evaluate significant differences between groups. Conversely, if the data deviated from normality, a nonparametric Kruskal–Wallis test combined with Dunn’s test was utilized to determine significant differences between groups. The specific statistical tests employed are detailed in the figure legends. Additionally, all statistical analyses and the creation of plots (e.g., histograms and scatter plots) were performed using the software GraphPad Prism 10 (version 10.6).

## 3. Results

### 3.1. Composition of the Prepared GSP

Table S4 shows that the concentrations of procyanidin, polyphenol, flavonoid, and polysaccharide in the prepared GSP were 10.40, 25.80, 3.56, and 0.39 g/100 g, respectively. Meanwhile, the Pb content was 0.12 mg/kg, and the aerobic bacterial count, the molds and yeasts count, and coliforms content were lower than 10 CFU/g, 10 CFU/g, and 0.3 MPN/g, respectively. Additionally, no Cd, Hg, As, DDT, HCH, PCNB, Salmonella, or *Staphylococcus aureus* were detected. All the detected indexes met the national food safety standards of China.

### 3.2. Growth Performance of Crisped Grass Carp

**Figure 1:**
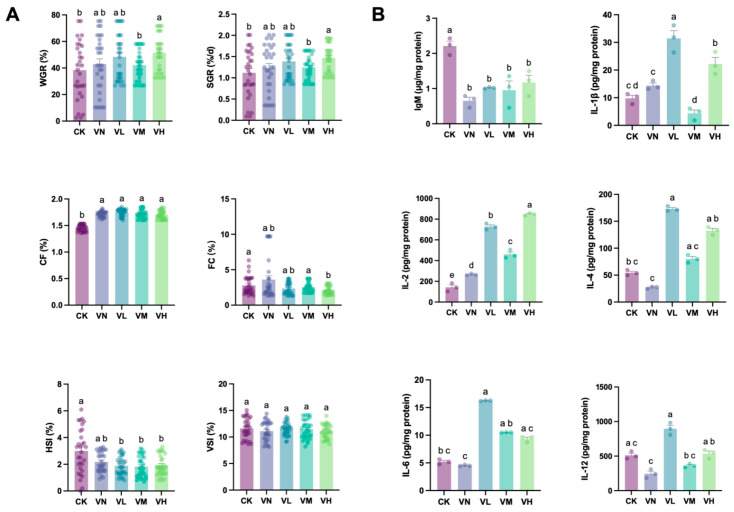
The growth parameters and the biochemical indexes of crisped grass carp. (**A**) the growth parameters; and (**B**) the immunoglobulin and Interleukins. Different lowercase letters indicate statistically significant differences between groups (Kruskal-Wallis test, *p* < 0.05).

Figure 1A shows that although WGR and SGR increased in the positive group (group VN) in comparison with the blank control (group CK), no significant difference was detected (*p* > 0.05). However, compared with groups CK and VN, an increase in WGR and SGR was determined in the GSP-supplemented groups (groups VL, VM, and VH), and a significant peak was observed in the high-GSP-supplemented group (group VH) (WGR = 420.74%, SGR = 1.73%, *p* < 0.05). Meanwhile, significant differences in WGR and SGR were monitored between the middle- and high-GSP-supplemented groups (groups VM and VH) (*p* < 0.05). Intriguingly, significant elevations in CF were also detected in group VN and the GSP-supplemented groups (groups VL, VM, and VH) in comparison with group CK (*p* < 0.05). Although a slight increase in FC was noted in group VN in comparison with group CK, a decrease was evident in the GSP-supplemented groups (groups VL, VM, and VH), with significant difference between group CK and the high-GSP-supplemented group (group VH) (*p* < 0.05). Additionally, the HSI decreased in group VN and the GSP-supplemented groups (groups VL, VM, and VH) in comparison with group CK. Moreover, significant differences in the HSI between group CK and the GSP-supplemented groups (group VL, VM, and VH) were observed (*p* < 0.05). However, no significant difference in the VSI was found between the groups.

### 3.3. Biochemical Indexes of Crisped Grass Carp

Figure 1B shows that the dominant IgM significantly decreased in group VN and the GSP-supplemented groups (groups VL, VM, and VH) in comparison with group CK (*p* < 0.05). Although a slight increase in IgM was found in the GSP-supplemented groups (groups VL, VM, and VH) in comparison with group VN, no statistically significant difference was determined (*p* > 0.05). Except for IL-2, which showed a significant elevation in group VN when compared with group CK (*p* < 0.05), no significant differences were observed between the other detected interleukins (IL-1*β*, IL-2, IL-4, IL-6, and IL-12) between the two groups (*p* > 0.05). Interestingly, these interleukins in the GSP-supplemented groups (groups VL, VM, and VH) mostly increased when compared with groups CK and VN, except IL-1*β* and IL-12 in the middle-GSP-supplemented group (group VM). Specifically, IL-1*β*, IL-4, IL-6, and IL-10 in the low-GSP-supplemented group (group VL) were significantly higher than those in groups CK and VN (*p* < 0.05).

### 3.4. Antioxidant Enzymes/Components of Crisped Grass Carp

**Figure 2:**
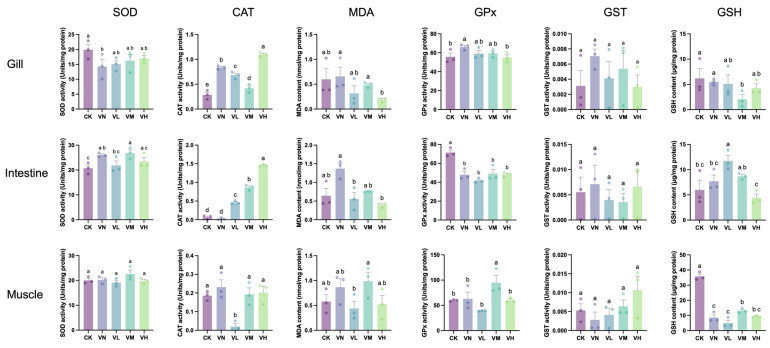
The levels of antioxidant enzymes/components in crisped grass carp. Different lowercase letters indicate statistically significant differences between groups (Kruskal-Wallis test, *p* < 0.05).

Figure 2 shows that a significant decrease in the SOD activity in the gills of group VN was observed when compared with group CK (*p* < 0.05); however, no significant difference in the GSP-supplemented groups (groups VL, VM, and VH) was determined in comparison with group CK (*p* > 0.05). Meanwhile, the SOD activities in the intestine and muscles displayed no significant difference between groups (*p* > 0.05), except SOD in the intestine between group CK and the middle-GSP-supplemented group (group VM). Remarkably, the CAT activities in the gills and intestine were significantly upregulated in the GSP-supplemented groups (groups VL, VM, and VH) in comparison with group CK (*p* < 0.05), especially with a relatively high daily supplement of GSP. Conversely, the CAT activities in the muscles of the low-GSP-supplemented group (group VL) were significantly lower than those of the other groups (*p* < 0.05). Additionally, a significant upregulation in GPx activities was observed in the gills of group VN when compared with group CK (*p* < 0.05), while this activity decreased with the daily supplementation of GSP, with a significant difference observed between group VN and the high-GSP-supplemented group (group VH) (*p* < 0.05). Moreover, the GPx activities in the intestine significantly decreased in group VN and the GSP-supplemented groups (groups VL, VM, and VH) in comparison with group CK (*p* < 0.05). To our surprise, no significant difference in GST activities was detected between groups across all tissues (*p* > 0.05). Furthermore, the MDA content in the gills and intestine of the high-GSP-supplemented (group VH) was significantly lower than that of group VN (*p* < 0.05). Similarly, the GSH content in the muscles of group VN and the GSP-supplemented groups (groups VL, VM, and VH) was significantly lower than that of group CK (*p* < 0.05). Therefore, dietary supplementation with GSP primarily affected the CAT activity in the gills and intestine, leading to a reduction in MDA content of these tissues. In addition, feeding a faba bean diet decreased muscle GSH content.

### 3.5. Histological Observation of Crisped Grass Carp

**Figure 3:**
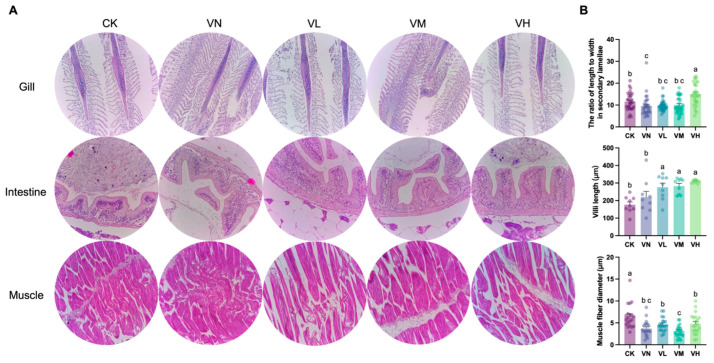
Tissue histological observation of crisped grass carp. (**A**) the representative images of the gill (200×), the intestine (400×), and the muscle (400×); and (**B**) quantitative analysis of histological changes, including the ratio of length to width in the secondary lamellae of the gills, villus length of the intestine, and the diameters of muscle fiber. Different lowercase letters indicate statistically significant differences between groups (Kruskal-Wallis test, *p* < 0.05).

The tissue histology (gills, intestine, and muscles) of crisped grass carp is shown in Figure 3A. The length-to-width ratio in the secondary lamellae of the gills in group VN significantly decreased when compared with group CK (*p* < 0.05), while it significantly increased in the high-GSP-supplemented group (group VH) (*p* < 0.05) (Figure 3B). Notably, the villi length increased in group VN and the GSP-supplemented groups (groups VL, VM, and VH) in comparison with group CK and was the longest in group VH (Figure 3B). Furthermore, a statistically significant difference was detected between group CK/VN and the GSP-supplemented groups (groups VL, VM, and VH) (*p* < 0.05). Interestingly, a significant decrease in muscle fiber diameter was observed between group CK and the other groups (*p* < 0.05) (Figure 3B), indicating that muscle depolymerization and breakdown were triggered due to feeding a faba bean diet. However, no significant difference in muscle fiber diameter was monitored between group VN and the GSP-supplemented groups (groups VL, VM, and VH).

### 3.6. Muscle Texture Properties of Crisped Grass Carp

**Figure 4:**
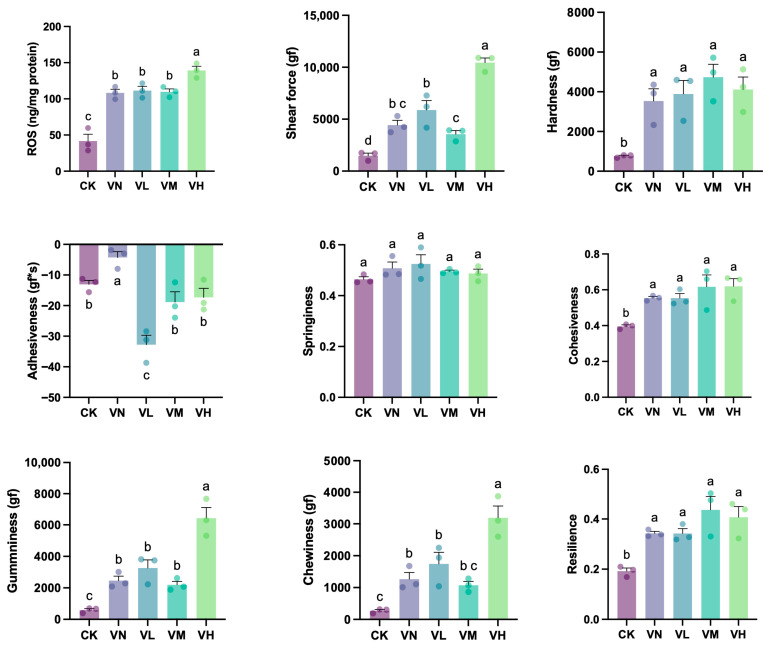
The muscle texture properties of crisped grass carp. Different lowercase letters indicate statistically significant differences between groups (Kruskal-Wallis test, *p* < 0.05).

A significant increase in muscle ROS content was determined in group VN and the GSP-supplemented groups (groups VL, VM, and VH) in comparison with group CK (*p* < 0.05) (Figure 4). Simultaneously, the muscle ROS content in the high-GSP-supplemented group (group VH) reached 139.2 ng/mg, which was significantly higher than that of the other groups (*p* < 0.05), suggesting that a relatively high dietary supplement of GSP stimulated muscle ROS production further. Additionally, the muscle shear force, gumminess, and chewiness in group VN were significantly elevated when compared with group CK (*p* < 0.05), and these indexes significantly increased with a relatively high daily supplementation of GSP compared to the indexes of group VN (*p* < 0.05), which reached 10,449, 6427, and 3190 gf in the high-GSP-supplemented group (group VH), respectively. Moreover, muscle hardness, cohesiveness, and resilience all increased in group VN and the GSP-supplemented groups (groups VL, VM, and VH) when compared with group CK, with a statistically significant difference (*p* < 0.05). Conversely, the muscle adhesiveness was the highest in group VN and the lowest in the low-GSP-supplemented group (group VL), which showed a significant difference when compared with the other groups (groups CK, VM, and VH) (*p* < 0.05). In addition, no significant difference in muscle springiness was observed between the groups (*p* > 0.05).

### 3.7. Muscle Amino Acids of Crisped Grass Carp

Although no significant difference in total amino acids was determined (*p* > 0.05), the total amino acids (TAAs) in the muscles of group VN increased when compared with group CK (Table 1). Nonetheless, the TAA contents in the GSP-supplemented groups (groups CK, VM, and VH) decreased and was the lowest in the low-GSP-supplemented group (group VL), which showed a significant difference with group VN (*p* < 0.05). Among the detected amino acids, Asp, Glu, Lys, and Leu were predominant since their contents exceeded the threshold of 1.00 g/100 g. Interestingly, the Pro contents in group VN and the GSP-supplemented groups (groups VL, VM, and VH) were increased when compared with group CK, with a significant difference between groups CK and VN/VL (*p* < 0.05). Remarkably, 13 amino acids (Asp, Phe, Tyr, Ala, Lys, Ser, Thr, Val, Leu, Met, Arg, His, and Ile) were the lowest in the low-GSP-supplemented group (group VL). Of these, 12 amino acids (except Tyr) revealed statistically significant differences with group VN, while ten amino acids (except Phe, Thr, and Ile) exhibited significant differences with the middle-GSP-supplemented group (group VM) (*p* < 0.05). Additionally, the Met content in the high-GSP-supplemented group (group VH) was also significantly higher than that of the low-GSP-supplemented group (group VL) (*p* < 0.05).

### 3.8. Muscle Fatty Acids of Crisped Grass Carp

Similar to amino acids, the total fatty acids (TFAs) in group VN increased when compared with group CK, without any significant differences (*p* > 0.05) (Table 2). Meanwhile, the TFA content decreased with the daily supplementation of GSP in comparison with group VN (*p* > 0.05); regardless, the TFA content in the high-GSP-supplemented group (group VH) was equivalent to that of group VN. Seven fatty acids exhibited significant differences between the five tested groups, including cis,cis-9,12-octadecadienoic acid (C18:2n6c), cis,cis,cis-9,12,15-octadecyltriaenoic acid (C18:3n3), cis,cis-11,14-eicosadienoic acid (C20:2n6), cis,cis,cis-8,11,14-eicosotrienic acid (C20:3n6), cis-5,8,11,14,17-eicosapentaenoic acid (C20:5n3), docosatetraenoic acid (C22:4n6), and cis-4,7,10,13,16,19-docosahexaenoic acid (C22:6n3). Of these, C20:5n3 in the middle-GSP-supplemented group (group VM) and C22:4n6 in group CK were undetected, and their contents displayed no significant difference between the other groups (*p* > 0.05). Additionally, C18:2n6c and C20:3n6 in the high-GSP-supplemented group (group VH) were significantly higher than C18:2n6c in the low-GSP-supplemented group (group VL) and C20:3n6 in group CK (*p* < 0.05), respectively. Moreover, C20:2n6 in group CK and the low-GSP-supplemented group (group VL) was significantly lower than that of group VN.

### 3.9. Muscle Safety of Crisped Grass Carp

Table S5 shows that the contents of Pb, Cd, Hg, and As in all tested groups were lower than 0.2, 0.05, 0.05, and 0.1 mg/kg, respectively. Simultaneously, no pesticide residues (DDT, HCH, and PCNB) and food pathogenic microbes (Salmonella and *Escherichia coli*) were detected in the muscles sampled from the tested groups; thus, the muscles of crisped grass carp with feeding inclusion GSPs were safe for consumption.

### 3.10. Intestine Microbiome of Crisped Grass Carp

**Figure 5:**
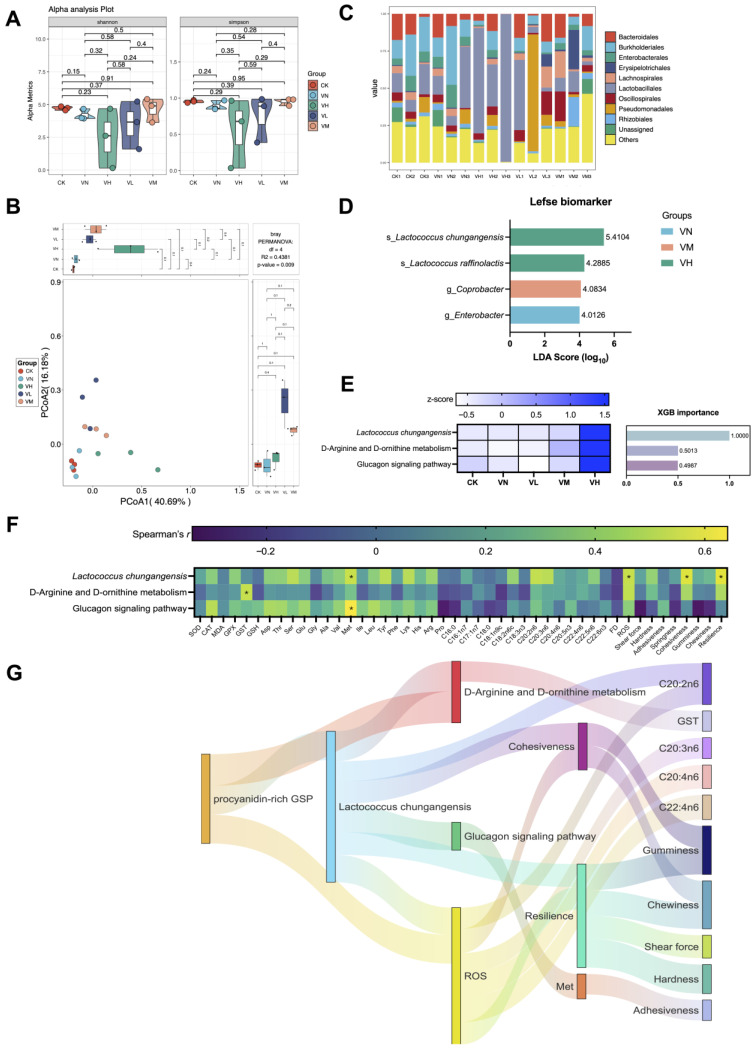
The intestinal microbiota of crisped grass carp. (**A**) alpha diversity based on nonparametric *t*-test (Shannon and Simpson indexes); (**B**) beta diversity based on PCoA; (**C**) top 10 bacterial taxa at the class level; (**D**) biomarker based on LEfSe; (**E**) potential key taxa and imputed functional profiles based on XGB; (**F**) Spearman’s correlation between the key taxa, the imputed functional profiles, and the muscle parameters. * indicates statistical significance (*p* < 0.05); and (**G**) Sankey plot based on significant relationships (Spearman’s *r* > 0.3 and *p* < 0.05) between the feed additives, the key bacterial taxa, the imputed functional profiles, and the muscle parameters.

Although no significant differences were identified between the groups regarding alpha diversity (Shannon and Simpson indexes) (*p* > 0.05) (Figure 5A), apparent compositional changes in the intestine microbiota were observed between the groups, with statistically significant differences (*p* < 0.05) (Figure 5B). Meanwhile, the classes Bacteroidales, Burkholderiales, Enterobacterales, Erysipelotrichales, Lachnospirales, Lactobacillales, Oscillospirales, Pseudomonadales, and Rhizobiales were the top eight predominant taxa due to their relative abundance accounting for the majority across classes (Figure 5C). Specifically, four biomarkers were screened out based on Lefse analysis. Of these, *Lactococcus chungangensis* and *L. raffinolactis* were associated with the high-GSP-supplemented group (group VH), *Coprobacter* was correlated with the middle-GSP-supplemented group (group VM), and *Enterobacter* was characteristic of group VN (Figure 5D). Moreover, *L. chungangensis* was recognized as the key taxa, which co-occurred with the key imputed functional profiles “D-Arginine and D-omithine metabolism” and “Glucagon signaling pathway” based on XGB analysis (Figure 5E). Remarkably, these key taxa and imputed functional profiles were highly expressed in the high-GSP-supplemented group (group VH) in comparison with the other groups. Notably, the key taxa and the imputed functional profiles exhibited significant correlations with GST, Met, ROS, cohesiveness, and resilience (*p* < 0.05) (Figure 5F).

### 3.11. Schematic Route of GSP Affecting Growth and Muscle Crispness of Crisped Grass Carp

The potential links between the keystone taxa, the imputed functional profiles, and the detected muscle indexes were established (Figure 5G). At the beginning, a relatively high daily supplement of GSP influenced *L. chungangensis* and demonstrated positive relationships with the imputed functional profiles related to “D-Arginine and D-omithine metabolism” (Spearman’s correlation coefficient *r* = 0.771, *p* = 0.001) and “Glucagon signaling pathway” (Spearman’s correlation coefficient *r* = 0.718, *p* = 0.004), which significantly contributed to ROS elevation in the muscles (Spearman’s *r* = 0.704, *p* = 0.005). Subsequently, the imputed functional profiles of “Glucagon signaling pathway” significantly influenced the Met content in the muscles (Spearman’s *r* = 0.642, *p* = 0.012), and the ROS level was well associated with cohesiveness (Spearman’s *r* = 0.629, *p* = 0.014) and resilience (Spearman’s *r* = 0.575, *p* = 0.027). Finally, these alterations were significantly conducive to the variations in muscle fatty acids (C20:2n6, C20:3n6, C20:4n6, and C22:4n6) and the modifications in muscle texture (gumminess, chewiness, shear force, hardness, and adhesiveness). These results suggested that the daily addition of GSPs enhanced muscle crispness via intestinal microbial mediation.

## 4. Discussion

### 4.1. Relatively High Daily Supplement of GSP Is Growth-Promoting for Crisped Grass Carp

Commonly, feeding inclusion plant-based ingredients is beneficial for nutritional availability and positively affects the growth performance of fishes [35,36,37]. For instance, the growth performance of *Megalobrama hoffmanni*, hybrid grouper (*Epinephelus lanceolatus* ♂ × *Epinephelus fuscoguttatus* ♀), and *Trachinotus ovatus* was substantially improved with the dietary addition of *Ampelopsis grossedentata* extract [38], *Panax notoginseng* extract [39], and *Crataegus monogyna* extract [40], respectively. In this study, the prepared GSP was a significant source of bioactive ingredients, as its procyanidin content surpassed 10%, which was significantly higher than that of the grape seeds globally (<2%) [41]. Based on the results shown, significant increases in the WGR and SGR of crisped grass carp were observed after supplemental feeding with GSP and reached their peak in the high-GSP-supplemented group, suggesting that dietary supplementation with GSP effectively elevated the growth performance of crisped grass carp. The growth-promoting effects of GSP might be ascribed to its ability of mitigating hepatic disorders [42], regulating gut function [43], and inhibiting oxidative stress [44], which has been verified in other fish or fish cells. Furthermore, an improvement in intestine histomorphology was usually observed with plant-based ingredients [4], which was closely linked to the growth performance of fish. According to histomorphology, an increase in villi length was conducive to nutrient absorption [45]. Interestingly, the villi length of the intestine in the GSP-supplemented groups significantly increased compared with the blank control and the positive group (*p* < 0.05). Simultaneously, a decrease in FC implied an elevation in the feed conversion ratio [12]. Consistently, a marked decrease in FC was determined with dietary supplementation with GSP, especially a relatively high daily supplement of GSP. Based on these results, the enhanced growth performance of crisped grass carp might be ascribed to the ameliorated intestine histomorphology caused by a relatively high daily supplement of GSP, which was well associated with nutrient absorption and transformation in crisped grass carp.

### 4.2. Relatively High Daily Supplement of GSP Facilitates Muscle Crispness

Previous evidence has shown that the faba bean-induced muscle crispness was mediated by ROS. A well-recognized view was that an increase in muscle ROS decreased myosin and actin contents, lowered myofibrillar calcium sensitivity, and reduced muscle fiber diameter, thus resulting in collagen turnover disruption [1]. In our study, the ROS content in the muscles significantly increased in the positive group in comparison with the blank control (*p* < 0.05), confirming that an increase in ROS content was essential for muscle crispness. Notably, the ROS level in the high-GSP-supplemented group was significantly higher than that of the positive group (*p* < 0.05), indicating that dietary supplementation with GSP stimulated ROS production further. Concurrently, the depolymerization and breakdown of muscle fibers, as well as a decline in muscle fiber diameters were both determined in the positive group and the GSP-supplemented groups when compared with the blank control, certifying that muscle crispness happened inevitably. In addition, the muscle texture was apparently different between the ordinary grass carp and the crisped grass carp, such as the chewiness, springiness, and gumminess [46]. Similarly, the muscle shear force, gumminess, chewiness, hardness, cohesiveness, and resilience increased in the positive group and the GSP-supplemented groups when compared with the blank control (*p* < 0.05). Of these, the muscle shear force, gumminess, and chewiness in the high-GSP-supplemented group were higher than those in the positive group, suggesting that a relatively high daily supplement of GSP caused more alterations in muscle crispness. Our former research had pointed out that a reduction in antioxidant capacity was responsible for ROS elevation, which resulted in muscle crispness [4]. Moreover, GSH played a crucial role in antioxidant defenses by participating in cellular redox reactions [47]. Notably, the GSH content in the muscles significantly decreased in the positive group and the GSP-supplemented groups in comparison with the blank control (*p* < 0.05), indicating a decrease in antioxidant capacity. In addition, dietary supplementation with GSP also affected the CAT activity in the gills and intestine, leading to a reduction in the MDA content of these tissues. These results proved that the daily supplementation of faba bean diet and GSP induced the tissue-specific redox imbalance. Furthermore, oxidative stress was normally accompanied with inflammatory reactions, which reflected the pathophysiology to some extent [48]. Remarkably, interleukins (e.g., IL-2) were increased in the positive group and the GSP-supplemented groups. Additionally, all detected interleukins in the high-GSP-supplemented group were higher than those in the positive group, which corroborated the higher ROS content in the same group. Studies have reported that dietary supplementation with grape seed extract at a dosage below 500 mg/kg contributed to inflammation reduction in animals [49,50,51]; however, if the dosage of grape seed extract surpassed 1000 mg/kg, inflammation response was provoked [52,53]. In line with prior research, our results supported that inflammatory reactions were triggered in crisped grass carp with a dosage of GSP of 1000 mg/kg, which was the effect of GSP and faba bean diet. Considering the abovementioned aspects, it was evident that a relatively high daily supplement of GSP elevated ROS content, triggered inflammatory response, and promoted muscle crispness in the aquaculture of crisped grass carp.

### 4.3. Relatively High Daily Supplement of GSP Affects Muscle Nutrition and Muscle Texture via Intestine Microbiota Mediation

Muscle amino acids and muscle fatty acids are well known to be the primary nutrition contributors of fish [54]. In our work, an increase in TAAs and TFAs was detected in the positive group, indicating that feeding a faba bean diet was conducive to both amino acid and fatty acid accumulation. Meanwhile, Pro significantly increased in the positive group, indicating that collagen regeneration occurred due to Pro participation in collagen biosynthesis [55]. However, a decrease in TAAs and TFAs was observed in the GSP-supplemented groups when compared with the positive group. This phenomenon could be mostly explained with a former study which reported that grape seeds participated in protein and lipid digestibility via regulating the in vivo metabolism [56,57]. Regardless of the decrease in amino acids and fatty acids with daily supplementation of GSP, their contents in the high-GSP-supplemented group were equivalent to those in the positive groups. Previous evidence has confirmed that intestine microbiota is closely related to nutrient metabolism and subsequently influenced the nutritional composition of muscles via intestine microbiota mediation [4]. Additionally, numerous studies have illustrated that grape seeds could ameliorate antimicrobial resistance and regulate intestine function [23,58]. In this study, significant compositional changes in the intestine microbiota were monitored between groups, indicating that feeding inclusion GSPs shaped the intestine microbiota. In fishes, a critical mechanism involving the alterations in intestine microbiota utilized probiotics as dietary supplementation with plant-based ingredients [59]. Furthermore, dietary supplementation with GSP also utilized probiotics, and *L. chungangensis* was the principal biomarker of the high-GSP-supplemented group due to its high expression. The literature has reported that *L. chungangensis* is well associated with amino acid and fatty acid metabolism [60,61]. In addition, our findings revealed that *L. chungangensis* exhibited significant correlations with “D-Arginine and D-omithine metabolism” and “Glucagon signaling pathway” based on imputed functional profiles. Moreover, *L. chungangensis* and the related functional profiles displayed substantial correlations with the ROS content, the altered amino acid (e.g., Met), and the muscle texture parameters (e.g., cohesiveness and resilience) based on Spearman’s correlation analysis. Integrating the abovementioned findings, we generated a Sankey diagram linking keystone taxa, inferred functional profiles, and muscle indexes. We observed that a relatively high daily supplement of GSP influenced *L. chungangensis* and affected pathways such as “D-Arginine and D-omithine metabolism” and “Glucagon signaling pathway“, subsequently increasing the muscle ROS content, and finally resulting in modifications in muscle nutrition (Met, C20:2n6, C20:3n6, C20:4n6, and C22:4n6) and muscle texture (gumminess, chewiness, shear force, hardness, and adhesiveness).

## 5. Conclusions

This study elucidates how feeding inclusion GSPs enhance the growth performance and muscle crispness of crisped grass carp. First, a growth-promoting effect was observed with a relatively high dietary supplement of GSP (1000 mg/kg), evidenced by the increase in WGR, SGR, and CF as well as the decrease in FC. Second, a relatively high daily supplement of GSP simultaneously facilitated muscle crispness, which could be attributed to an increased ROS content. Last, it modulated the intestine microbiota, influencing *L. chungangensis* and subsequently the nutritional quality as well as muscle texture of crisped grass carp. Our findings concluded that a relatively high daily supplement of GSP was beneficial for the aquaculture of crisped grass carp.

## Figures and Tables

**Table 1:** The contents of muscle amino acids of crisped grass carp.

Item	Amino Acid (g/100 g FW)	CK	VN	VL	VM	VH
DAA	Asp	1.46 ± 0.04 ^ab^	1.58 ± 0.08 ^a^	1.22 ± 0.02 ^b^	1.53 ± 0.10 ^a^	1.47 ± 0.05 ^ab^
	Glu	2.53 ± 0.08 ^a^	2.67 ± 0.14 ^a^	2.11 ± 0.09 ^a^	2.66 ± 0.14 ^a^	2.49 ± 0.04 ^a^
	Phe	0.62 ± 0.05 ^a^	0.65 ± 0.03 ^a^	0.50 ± 0.01 ^b^	0.60 ± 0.04 ^ab^	0.56 ± 0.02 ^ab^
	Gly	0.67 ± 0.03 ^a^	0.82 ± 0.06 ^a^	0.68 ± 0.01 ^a^	0.69 ± 0.04 ^a^	0.67 ± 0.03 ^a^
	Tyr	0.58 ± 0.03 ^ab^	0.60 ± 0.03 ^ab^	0.54 ± 0.01 ^b^	0.62 ± 0.02 ^a^	0.59 ± 0.01 ^ab^
	Ala	1.02 ± 0.02 ^ab^	1.13 ± 0.04 ^a^	0.88 ± 0.03 ^b^	1.07 ± 0.03 ^a^	1.02 ± 0.03 ^ab^
SAA	Lys	1.32 ± 0.09 ^ab^	1.44 ± 0.10 ^a^	1.09 ± 0.05 ^b^	1.42 ± 0.07 ^a^	1.36 ± 0.03 ^ab^
	Pro	0.48 ± 0.06 ^b^	0.80 ± 0.12 ^a^	0.98 ± 0.01 ^a^	0.85 ± 0.10 ^ab^	0.73 ± 0.11 ^ab^
	Ser	0.56 ± 0.02 ^ab^	0.62 ± 0.03 ^a^	0.51 ± 0.01 ^b^	0.62 ± 0.02 ^a^	0.59 ± 0.02 ^ab^
	Thr	0.62 ± 0.02 ^ab^	0.68 ± 0.03 ^a^	0.55 ± 0.01 ^b^	0.66 ± 0.04 ^ab^	0.63 ± 0.02 ^ab^
BAA	Val	0.70 ± 0.02 ^ab^	0.74 ± 0.04 ^a^	0.59 ± 0.03 ^b^	0.72 ± 0.04 ^a^	0.70 ± 0.03 ^ab^
	Leu	1.20 ± 0.05 ^ab^	1.26 ± 0.07 ^a^	1.04 ± 0.02 ^b^	1.22 ± 0.09 ^a^	1.17 ± 0.03 ^ab^
	Met	0.40 ± 0.02 ^ab^	0.41 ± 0.02 ^a^	0.33 ± 0.01 ^b^	0.42 ± 0.04 ^a^	0.41 ± 0.02 ^a^
	Arg	0.85 ± 0.07 ^ab^	0.93 ± 0.05 ^a^	0.76 ± 0.02 ^b^	0.92 ± 0.04 ^a^	0.87 ± 0.03 ^ab^
	His	0.29 ± 0.03 ^ab^	0.34 ± 0.02 ^a^	0.25 ± 0.01 ^b^	0.32 ± 0.02 ^a^	0.30 ± 0.01 ^ab^
	Ile	0.68 ± 0.04 ^ab^	0.73 ± 0.03 ^a^	0.60 ± 0.02 ^b^	0.69 ± 0.06 ^ab^	0.64 ± 0.02 ^ab^
TAA		13.98 ± 0.65 ^ab^	15.40 ± 0.71 ^a^	12.63 ± 0.28 ^b^	15.01 ± 0.87 ^a^	14.20 ± 0.27 ^ab^
EAA/TAA (%)		0.37	0.36	0.34	0.36	0.36
DAA/TAA (%)		0.49	0.48	0.47	0.48	0.48
SAA/TAA (%)		0.21	0.23	0.25	0.24	0.23
BAA/TAA (%)		0.29	0.29	0.28	0.29	0.29

Note: EAA represents essential amino acids; DAA represents delicious amino acids; SAA represents sweet amino acids; BAA represents bitter amino acids; and FW represents fresh weight. Different lowercase letters indicate statistically significant differences between groups (Kruskal Wallis test, *p* < 0.05).

**Table 2:** The contents of muscle fatty acids of crisped grass carp.

Fatty Acids (g/100 g FW)	CK	VN	VL	VM	VH
Hexadecanoic acid (C16:0)	0.112 ± 0.009 ^a^	0.114 ± 0.021 ^a^	0.123 ± 0.004 ^a^	0.103 ± 0.017 ^a^	0.114 ± 0.009 ^a^
Cis-9-hexadecane-1-enoic acid (C16:1n7)	0.013 ± 0.003 ^a^	0.011 ± 0.004 ^a^	0.012 ± 0.002 ^a^	0.009 ± 0.002 ^a^	0.013 ± 0.003 ^a^
Cis-10 heptadecaenoic acid (C17:1n7)	0.010 ± 0.002 ^a^	0.008 ± 0.004 ^a^	0.016 ± 0.003 ^a^	0.013 ± 0.004 ^a^	0.013 ± 0.002 ^a^
Octadecanoic acid (C18:0)	0.051 ± 0.006 ^a^	0.056 ± 0.009 ^a^	0.063 ± 0.004 ^a^	0.057 ± 0.009 ^a^	0.058 ± 0.003 ^a^
Cis-9-octadecenoic acid (C18:1n9c)	0.012 ± 0.002 ^a^	0.014 ± 0.002 ^a^	0.012 ± 0.001 ^a^	0.012 ± 0.001 ^a^	0.012 ± 0.001 ^a^
Cis,cis 9,12-octadecadienoic acid (C18:2n6c)	0.065 ± 0.007 ^ab^	0.097 ± 0.015 ^ab^	0.063 ± 0.007 ^b^	0.067 ± 0.003 ^ab^	0.077 ± 0.003 ^a^
Cis,cis,cis-9,12,15-octadecyltriaenoic acid (C18:3n3)	0.005 ± 0.001 ^ab^	0.006 ± 0.001 ^a^	0.001 ± 0.001 ^ab^	0.001 ± 0.001 ^b^	0.003 ± 0.002 ^ab^
Cis,cis 11,14-eicosadienoic acid (C20:2n6)	0.004 ± 0.002 ^b^	0.009 ± 0.001 ^a^	0.002 ± 0.002 ^b^	0.004 ± 0.002 ^ab^	0.008 ± 0.001 ^ab^
Cis,cis,cis-8,11,14-eicosotrienic acid (C20:3n6)	0.011 ± 0.001 ^b^	0.014 ± 0.001 ^ab^	0.012 ± 0.001 ^ab^	0.013 ± 0.001 ^ab^	0.014 ± 0.001 ^a^
Cis-5,8,11,14-eicosatetraenoic acid (C20:4n6)	0.058 ± 0.007 ^a^	0.065 ± 0.007 ^a^	0.072 ± 0.01 ^a^	0.062 ± 0.002 ^a^	0.071 ± 0.003 ^a^
Cis-5,8,11,14,17-eicosapentaenoic acid (C20:5n3)	0.007 ± 0.001 ^a^	0.002 ± 0.002 ^ab^	0.002 ± 0.002 ^ab^	0 ^b^	0.006 ± 0.003 ^ab^
Docosatetraenoic acid (C22:4n6)	0 ^b^	0.005 ± 0.002 ^ab^	0.005 ± 0.003 ^ab^	0.002 ± 0.002 ^ab^	0.007 ± 0.002 ^a^
Docosapentaenoic acid (C22:5n6)	0.031 ± 0.003 ^a^	0.043 ± 0.006 ^a^	0.033 ± 0.001 ^a^	0.034 ± 0.001 ^a^	0.039 ± 0.002 ^a^
Cis-4,7,10,13,16,19-docosahexaenoic acid (C22:6n3)	0.042 ± 0.007 ^a^	0.030 ± 0.005 ^ab^	0.030 ± 0.001 ^b^	0.030 ± 0.004 ^ab^	0.030 ± 0.004 ^ab^
TFA	0.423 ± 0.046 ^a^	0.474 ± 0.076 ^a^	0.446 ± 0.004 ^a^	0.407 ± 0.031 ^a^	0.463 ± 0.019 ^a^

Note: Different lowercase letters indicate statistically significant differences between groups (Kruskal Wallis test, *p* < 0.05).

## Data Availability

All datasets collected and analyzed in the study are available from the corresponding author upon reasonable request.

## Supplementary Materials

The following supporting information can be downloaded at: https://www.mdpi.com/article/10.3390/ani16020251/s1, Table S1: The composition of the basal feed pellets “1038 Original Pond Particles”; Table S2: The composition of the faba bean diet; Table S3: The rearing conditions during the acclimation and feeding periods; Table S4: The material composition of the prepared GSP; Table S5: Muscle safety inspection of crisped grass carp.
