# Supplementary material for: High Dietary Supplementation of Procyanidin-Rich Grape Seed Powders Enhances the Growth Performance and Muscle Crispness of Crisped Grass Carp
Source: Animals (Basel). 2026 Jan 14;16(2):251. doi: 10.3390/ani16020251 (PMC12837870; doi:10.3390/ani16020251)
Supplement: Supplementary file 1 [file animals-16-00251-s001.zip › animals-4052101-supplementary.pdf]

Supplementary Material

Article

High Dietary Supplementation of Procyanidin-Rich Grape Seed Powders Enhances the Growth Performance and Muscle Crispness of Crisped Grass Carp

Ziqiu Peng [1,†], Qiuwen Tang [2,†], Haojun Liang [2], Xiaoyi Zhang [1], Xiaoye Wang [1], You Li [3], Ping Ding [1], Yongzhan Mai [4,*] and Xuesong Wang [1,*]

[1] Key Laboratory of Chinese Medicinal Resource from Lingnan, Ministry of Education, Institute of Medical Plant Physiology and Ecology, School of Pharmaceutical Sciences, Guangzhou University of Chinese Medicine, Guangzhou 510006, China; lluviapeng0928@163.com (Z.P.); shirleyzhang0111@163.com (X.Z.); 15298011853@163.com (X.W.); dingping19650320@126.com (P.D.)

[2] Guangdong Provincial Key Laboratory of Chemical Measurement and Emergency Test Technology, Guangdong Provincial Engineering Research Center for Ambient Mass Spectrometry, Institute of Analysis, Guangdong Academy of Sciences (China National Analytical Center, Guangzhou), 100 Xianlie Middle Road, Guangzhou 510070, China; 15307603376@163.com (Q.T.); 15362158017@163.com (H.L.)

[3] School of Chemistry, Chemical Engineering and Materials Science, Shandong Normal University, Jinan 250014, China; 18862737011@163.com

[4] Scientific Observing and Experimental Station of Fishery Resources and Environment in the Middle and Lower Reaches of Pearl River, Key Laboratory of Prevention and Control for Aquatic Invasive Alien Species, Fishery Ecological Environment Monitoring Center of Pearl River Basin, Ministry of Agriculture and Rural Affairs, Guangdong Provincial Key Laboratory of Aquatic Animal Immunology and Sustainable Aquaculture, Pearl River Fisheries Research Institute, Chinese Academy of Fishery Sciences, Guangzhou 510380, China

* Correspondence: yongzhanmai@prfri.ac.cn (Y.M.); wangxs@caiqgba.org.cn or wlwxs57813@gzucm.edu.cn (X.W.); Tel./Fax: +86-2084113220 (X.W.)

† These authors contributed equally to this work.

Table S1. The composition of the basal feed pellets “1038 Original Pond Particles”.

Item	Content (%)
Protein	28–40
Crude fat	≥ 3
Crude fibre	≤ 10
Crude ash	≤ 12
Moisture	≤ 10
Total phosphorus	≥ 1.0
Lysine	≥ 1.65
Ca	0.5–2.0
NaCl	0.3–1.2

Table S2. The composition of the faba bean diet.

Item	Content (%)
Crude Protein	28.15
Crude fat	3.02
Crude fibre	30.14
Crude ash	4.31
Moisture	7.62
Lysine	2.48

Table S3. The rearing conditions during the acclimation and feeding periods.

Item	Concentration
Dissolved oxygen (DO)	8.2 ± 0.3 mg/L
pH	7.4 ± 0.2
Water hardness (expressed as $CaCO_3$)	34.8 ± 0.5 mg/L
Salinity	0.10 ± 0.03‰
Nitrate nitrogen (NO_3-N)	1.4 ± 0.1 mg/L
Temperature	25 ± 0.5℃

Table S4. The material composition of the prepared GSP.

Category	Index	Content
Bioactive constituents	Procyanidine (g/100 g)	10.40 ± 0.12
	Polyphenol (g/100 g)	25.80 ± 2.13
	Flavonoid (g/100 g)	3.56 ± 0.27
	Polysaccharide (g/100 g)	0.39 ± 0.02
Heavy metals	Pb (mg/kg)	0.12 ± 0.01
	Cd (mg/kg)	ND
	Hg (mg/kg)	ND
	As (mg/kg)	ND
Pesticide residues	DDT (mg/kg)	ND
	HCH (mg/kg)	ND
	PCNB (mg/kg)	ND
Microbial contents	Salmonella (25 g)	ND
	Staphylococcus aureus (25 g)	ND
	Aerobic bacterial count (CFU/g)	< 10
	Moulds and Yeasts (CFU/g)	< 10
	Coliforms (MPN/g)	< 0.3

Table S5. Muscle safety inspection of crisped grass carp.

Category	Items	CK	TL	TM	TH
Heavy metals	Pb (mg/kg)	< 0.2	< 0.2	< 0.2	< 0.2
	Cd (mg/kg)	< 0.05	< 0.05	< 0.05	< 0.05
	Hg (mg/kg)	< 0.05	< 0.05	< 0.05	< 0.05
	As (mg/kg)	< 0.1	< 0.1	< 0.1	< 0.1
Pesticide residues	DDT (mg/kg)	-	-	-	-
	HCH (mg/kg)	-	-	-	-
	PCNB (mg/kg)	-	-	-	-
Food pathogenic microbes	Salmonella (CFU/mL)	-	-	-	-
	Escherichia coli (CFU/mL)	-	-	-	-
